# Supplementary material for: Causal Relationship between Aging and Anorexia Nervosa: A White-Matter-Microstructure-Mediated Mendelian Randomization Analysis
Source: Biomedicines. 2024 Aug 16;12(8):1874. doi: 10.3390/biomedicines12081874 (PMC11351342; doi:10.3390/biomedicines12081874)
Supplement: Supplementary file 1 [file biomedicines-12-01874-s001.zip › Supplementary Figure/Figure S8/Leaveoneout plot of MR analysis for TL to ubm-b-1935.pdf]

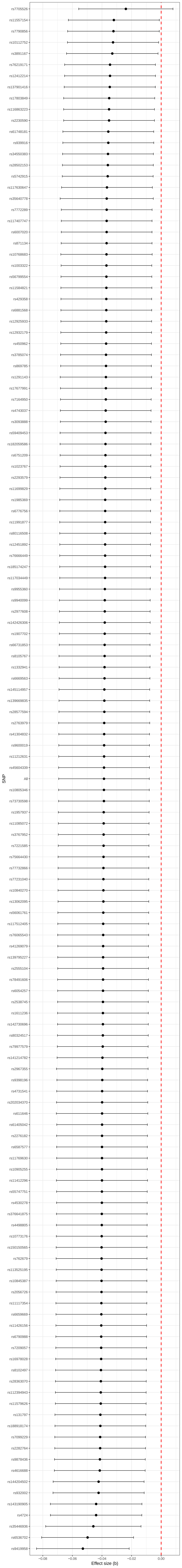

rs7705526
rs11557154
rs7790856
rs10112752
rs3891167
rs76219171
rs12412214
rs137901416
rs17803849
rs116863223
rs2230590
rs61748181
rs939916
rs34550383
rs28502153
rs5742915
rs117630647
rs35640778
rs7772289
rs117407747
rs6007020
rs871134
rs10768683
rs1003322
rs56799554
rs11584821
rs429358
rs6881568
rs12925933
rs12932179
rs450962
rs3785074
rs869785
rs1291143
rs17677991
rs7164950
rs4743037
rs3093888
rs59409453
rs182059586
rs6751209
rs1023767
rs2293579
rs11699829
rs1985369
rs6776756
rs11991877
rs80116508
rs12451892
rs76666449
rs185174247
rs117034449
rs9955360
rs9940099
rs2977608
rs142426306
rs1907702
rs66731853
rs8105767
rs1332941
rs6669563
rs145114957
rs139669835
rs28577594
rs2763979
rs41304832
rs9600019
rs11212631
rs45604339
All
rs10805346
rs73730598
rs1957937
rs11085072
rs3767952
rs7221585
rs75664430
rs77732866
rs77231040
rs10840270
rs13062095
rs56061761
rs117512405
rs76065543
rs41269079
rs139795227
rs2555104
rs78491606
rs6054257
rs2538745
rs1611236
rs142730696
rs80324517
rs79977579
rs141214782
rs2967355
rs9398196
rs4731541
rs202034370
rs611646
rs61405042
rs2276182
rs6587577
rs11769630
rs10905255
rs11412296
rs55747751
rs4530278
rs376641875
rs4498805
rs10773176
rs150150565
rs762679
rs113525195
rs10845387
rs2056726
rs11117354
rs6659669
rs11426156
rs6790988
rs7209057
rs16978028
rs8102497
rs28363070
rs112394943
rs11579626
rs131797
rs188918174
rs7099229
rs2282764
rs9878436
rs4616688
rs144204502
rs932002
rs143190905
rs4724
rs35446936
rs6536702
rs9419958
SNP
−0.08
−0.06
−0.04
−0.02
0.00
Effect size (b)
